# *Streptococcus suis* disrupts the blood–brain barrier through inducing ubiquitin–proteasome-mediated degradation of KAT2A

**DOI:** 10.1186/s13567-026-01736-8

**Published:** 2026-03-25

**Authors:** Yuqing Li, Hang Yin, Tao Zhang, Shiqi Lang, Tianning Yang, Zhiwei Li, Lianci Peng, Rendong Fang

**Affiliations:** Joint International Research Laboratory of Animal Health and Animal Food Safety, College of Veterinary Medicine, Southwest University, No.2 Tiansheng Road, Beibei District, Chongqing, 400715 China

**Keywords:** *Streptococcus suis*, KAT2A, blood–brain barrier (BBB), necroptosis

## Abstract

*Streptococcus suis* (*S. suis*) induces host cell death and has the ability to invade the blood–brain barrier (BBB). However, *S. suis*-induced BBB disruption has not been completely elucidated. In this study, we focused on the regulatory role of lysine acetyltransferase 2A (KAT2A) on BBB, which is a key regulator of cell death. We found that *S. suis* strain SC19 induces cell death in human cerebral microvascular endothelial cell line D3 (hCMEC/D3) by membrane disruption and LDH release. During SC19 infection, KAT2A protein expression was markedly reduced but its mRNA expression was not affected. Further study demonstrated that SC19-induced KAT2A reduction was though ubiquitin–proteasome-mediated protein degradation pathway. KAT2A inhibition using significantly exacerbated SC19-induced cell permeability disruption by transwell infection model. Consistently, KAT2A knockdown and pharmacological inhibition significantly aggravated SC19-induced downregulation of tight junction proteins including ZO-1 and occludin, whereas KAT2A overexpression partially restored their levels. Similarly, KAT2A inhibition aggravated SC19-induced downregulation of ZO-1 in mice brain and increased mice death rate, indicating the protective role of KAT2A on BBB. Mechanistically, KAT2A was found to regulate necroptosis in hCMEC/D3. KAT2A knockdown aggravated necroptosis and upregulated phosphorylation of RIPK1, whereas RIPK1 inhibitor Nec-1 rescued SC19-induced necroptosis, indicating that KAT2A regulates RIPK1-dependent necroptosis during SC19 infection. Furthermore, quantitative proteomic analysis identified a network of putative KAT2A-mediated downstream targets and pathways that contribute to BBB integrity. Collectively, these findings reveal the underlying mechanism by which SC19 disrupts BBB through inducing KAT2A-mediated necroptosis and provide a potential therapeutic target for the treatment of bacterial meningitis.

**Supplementary Information:**

The online version contains supplementary material available at 10.1186/s13567-026-01736-8.

## Introduction

*Streptococcus suis* (*S. suis*), a major zoonotic pathogen, causes meningitis and septicemia in pigs, leading to significantly economic losses in the swine industry [1]. Importantly, human cases of *S. suis* infection have been reported in certain geographical regions, most notably in parts of Southeast Asia, which pose a significant threat to populations with close contact with pigs or pork products [2–4]. *S. suis* typically colonizes in the upper respiratory tract, especially the tonsils, and the gastrointestinal tract of pigs, nevertheless it can cause severe systemic infection through disrupting mucosal barriers following by entering the bloodstream [1, 5]. Upon entering the blood circulation, *S. suis* can disseminate to the central nervous system by crossing the blood–brain barrier (BBB), thereby inducing meningitis [6]. The development and progression of meningitis generally rely on the pathogen’s ability to survive in the bloodstream and to penetrate the BBB, which are mediated by bacterial virulence factors, host brain endothelial cell responses and the inflammatory cascade [7]. Some virulence factors of *S. suis*, such as enolase [8], RTX family exoprotein A (RfeA) [9] and suilysin (sly) [10], have been identified as inducing cell death in human brain microvascular endothelial cells (HBMECs), including apoptosis and pyroptosis [11–13]. Such cell death contributes to tight-junction disruption and endothelial barrier dysfunction of BBB, thereby promoting the development of meningitis. In addition, upon microbial infection, some host factors have been found to play important roles in the BBB. Our previous study has shown that NLRP3 inflammasome is involved in *S. suis*-induced BBB disruption. The host factor ARF6 facilitates *S. suis*-triggered apoptosis in HBMECs [10]. However, whether other potential host factors mediate *S. suis*-induced BBB disruption remains to be elucidated.

Lysine acetyltransferase 2 A (KAT2A) also known as GCN5, is an evolutionarily conserved histone acetyltransferase, regulating various physiological and pathological processes, such as protein post-translational modifications, cellular metabolism, cancer diseases [14–16] and cell proliferation [17]. The deletion of KAT2A in embryonic stem cells causes embryonic lethality in mice and elevates cell apoptosis [18]. KAT2A acts as an acetyltransferase to catalyze histone succinylation and lactylation, modulating tumor development [14, 19]. Recent studies have reported that KAT2A modulates ferroptosis through distinct disease-specific mechanisms, which involve its enzymatic activity and modulation of specific downstream targets across different pathological situation including colorectal cancer, diabetic nephropathy and diabetic cardiomyopathy [20–22]. In addition, upon viral infection, KAT2A exerts critical effects on viral replication through epigenetic modulation of viral genetic material and post-translational modification of viral proteins [23–25]. However, whether KAT2A plays a regulatory role in bacterial infections remains to be elucidated.

In this study, we investigated the regulatory role of KAT2A in *S. suis*-induced BBB disruption. Our study reveals a protective role of KAT2A in maintaining endothelial viability and BBB homeostasis during *S. suis* infection, which provides a potential therapeutic target for *S. suis* meningitis.

## Materials and methods

### Ethic statements

Wild-type (WT) C57BL/6 mice were purchased from Chongqing Enswell Biotechnology Co., Ltd. All the mice were maintained under specific pathogen-free (SPF) conditions before being used at 8–10 weeks of age. All the animal experiments were approved by the Southwest University Ethics Committee, Chongqing, China (IACUC-20231215–02).

### Bacterial strain

SC19, a prevalent *S. suis* strain provided by Professor Xiangru Wang (College of Veterinary Medicine, Huazhong Agricultural University), was cultured in Todd-Hewitt broth (THB; OXOID, CM0189) supplemented with 10% fetal bovine serum (FBS; Zhejiang Tianhang Biotechnology Co., Ltd., cat. no. 23022–8615) at 37 °C. Bacterial growth was monitored by measuring optical density at 600 nm (OD_600_).

### Cell culture and bacterial infection

The human cerebral microvascular endothelial cell line (hCMEC/D3, JNO-H0520, Jennio Biotech) cells were maintained in DMEM (Gibco, C11995500BT) supplemented with 10% FBS (ExCell Bio, FSP500) at 37 °C in a humidified incubator with 5% CO₂. Cells were infected with SC19 at a multiplicity of infection (MOI) of 10 for 1, 2, or 4 h. After infection, the supernatants and cell lysates were collected for assay as described below. Additional chemicals, including chloroquine (CQ; MedChemExpress, HY-17589A; 50 μM, 2 h), MG-132 (MedChemExpress, HY-13259; 50 μM, 2 h), necrostatin-1 (Nec-1; MedChemExpress, HY-15760; 10 μM, 1 h), and butyrolactone 3 (MB-3; Sigma-Aldrich, M2449; 50 μM, 24 h) were added to cells prior to infection for 1, 2 or 24 h.

### Propidium iodide (PI) staining

hCMEC/D3 cells were seeded in 6-well plates and infected with SC19 as described above. After 4 h infection, cells were stained with hoechst33342 and propidium iodide (PI) (KeyGEN BioTECH, KGA1803-100) according to the manufacturer’s instructions, and then observed under a fluorescence microscope (Olympus, Tokyo, Japan).

### Flow cytometry

hCMEC/D3 cells were seeded in 6-well plates and infected with SC19 as described above. After 4 h of infection, adherent cells were gently detached using trypsin solution without EDTA and collected by centrifugation at 300 × *g* for 5 min at 4 °C. The cell pellet was washed twice with cold PBS (300 × *g*, 5 min each) and then resuspended in 500 μL of 1 × Binding Buffer provided in the Annexin V-FITC/PI Apoptosis Detection Kit (KeyGEN BioTECH, KGA1102-50). Subsequently, 5 μL Annexin V-FITC and 5 μL PI were added to each sample and mixed gently. Then, cell suspension was incubated for 10 min at room temperature in the dark, and analyzed within 1 h. Fluorescence signals were determined using a NovoCyte Flow Cytometer (Agilent, USA) and analyzed using NovoExpress (Agilent, USA). Three replicate wells for each of the above treatments were analyzed.

### Detection of lactate dehydrogenase (LDH)

Cells were infected with SC19 as described above in 6- or 96-well plates for 4 h. To generate 100% LDH release, 10% lysis solution was added into non-infected cells defined as positive control group. Cell culture medium was used to be defined as background group. After infection, supernatants were collected for the measurement of the level of LDH release using LDH Cytotoxicity Assay Kit (Promega, G1780) according to the manufacturer’s instructions. Briefly, 50 μL of these supernatants were mixed with 50 μL of CytoTox 96^®^ Reagent at room temperature for 30 min, followed by the addition of 50 μL of stop solution. Finally, the absorbance was recorded at 490 nm using a microplate reader (Bio-Rad, Japan). The LDH release (%) was calculated as follows:

$${\text{LDH release }}\left( \% \right)\, = \,\left( {{\mathrm{OD}}_{{{\mathrm{sample}}}} - {\mathrm{OD}}_{{{\mathrm{background}}}} } \right)/\left( {{\mathrm{OD}}_{{{\mathrm{positive}}}} - {\mathrm{OD}}_{{{\mathrm{background}}}} } \right){\text{ }}*{\text{ 1}}00\%$$

### Western blotting

Cells and homogenized brain tissues were lysed using ice-cold cell lysis buffer (Beyotime, P0013F) or RIPA buffer (MedChemExpress, HY-K1001) supplemented with a protease inhibitor (Beyotime, P1005). Then, the lysates were mixed with loading buffer and boiled at 100 °C for 10 min followed by being subjected to 8% or 12% SDS-PAGE gels. Subsequently, the proteins were transferred to polyvinylidene difluoride (PVDF) membranes (Merck Millipore, IPVH00010) by electroblotting. After blocking with 5% nonfat dry milk for 2 h at room temperature, membranes were immunoblotted with the indicated primary antibodies (Abs) overnight and then incubated with the appropriate HRP-conjugated secondary Abs (Beyotime, A0208 and A0216). Finally, protein bands were detected with an enhanced chemiluminescence (ECL) detection reagent (Biosharp, China) and visualized using a ChemiDoc Imaging System (Bio-Rad, USA). ImageJ was used to quantify relative expression levels of proteins against β-actin. The primary Abs contained anti-ZO-1 (Proteintech, 21,773–1-AP), anti-KAT2A (Proteintech, 66,575-1-Ig), anti-Occludin (Proteintech, 27,260–1-AP), anti-RIPK1 (Proteintech, 29,932-1-AP), anti-Phospho-RIPK1(Ser166) (Proteintech, 28,252-1-AP), anti-Phospho-RIPK1(Ser161) (Proteintech, 66,854–1-AP), anti-ubiquitin (Proteintech, 10,201-2-AP), anti-Caspase-8 (Proteintech, 13,423-1-AP), anti-β-actin (ABclonal, AC026), anti-AKR1B1 (Abmart, M63224), anti-Podocalyxin-like 1 (UpingBio, YP-Ab-13820), anti-A2AP (UpingBio, YP-mAb-05308), anti-GPAM (UpingBio, YP-mAb-18783).

### Quantitative real time polymerase chain reaction (RT-qPCR)

Total RNA was extracted using the FastPure Complex Tissue/Cell Total RNA Isolation Kit (Vazyme, RC113) according to the manufacturer’s instructions. Subsequently, 1 μg of total RNA was reverse transcribed into cDNA using the HiScript III RT SuperMix for qPCR (Vazyme, R323). qPCR was performed using ChamQ Blue Universal SYBR qPCR Master Mix (Vazyme, Q312) on a CFX96 Real-Time PCR Detection System (Bio-Rad, USA). Relative gene expression levels were normalized to GAPDH expression. The primer sequences were as follows: KAT2A, forward 5’-TACTTCCTCACCTACGCCGA-3’ and reverse 5’-CTTGGGCACCTTGATGTCCT-3’; GAPDH, forward 5’-GGAGCGAGATCCCTCCAAAAT-3’ and reverse 5’-GGCTGTTGTCATACTTCTCATGG-3’.

### Co-immunoprecipitation (Co-IP) assay

hCMEC/D3 cells were prepared and infected with SC19 as described above. After 2 and 4 h infection, cells were lysed in ice-cold cell lysis buffer on ice and then centrifuged at 12 000 rpm for 15 min at 4 °C. Next, supernatants were collected and incubated overnight at 4 °C with anti-KAT2A. Subsequently, Protein A + G agarose beads (Beyotime, P2055) were added to the mixture and incubated for 4 h at 4 °C. The beads were washed with ice-cold PBS three times, and resuspended in 2 × SDS loading buffer. Finally, collected beads were boiled at 100 °C for 10 min and subjected to immunoblotting analysis for immunocomplexes using anti-KAT2A and anti-ubiquitin.

### Endothelial cell permeability assay

Endothelial cell permeability was measured using transwell model. Cells were cultured on Transwell-Clear inserts with a pore diameter of 3 μm (Corning Costar, USA) for 7–10 days until the transepithelial electrical resistance (TEER) reached a stable value exceeding 200 Ω.cm^2^ [26]. Subsequently, cells in the upper chamber were infected with SC19 at an MOI of 10 and incubated at 37 °C for 1, 2, 3 and 4 h. In addition, MB-3 was added to inhibit KAT2A prior to infection for 24 h. After infection, the value of TEER (Ω.cm^2^) was detected by the Millicell-ERS electrical resistance system to evaluate its integrity as previously described [27].

### RNA interference and KAT2A overexpression

Cells were transfected with KAT2A siRNA or a negative control (NC) siRNA for 48 h using Lipofectamine 3000 (Thermo Fisher Scientific, USA) following the manufacturer’s protocol. The siRNA sequences used in the present study were as follows: KAT2A siRNA-1 (Sangon Biotech, 5’-CCUCGAAUGAGCAGGUCAA-3’), KAT2A siRNA-2 (Sangon Biotech, 5’-CAGACACCAAGCAGGUCUA-3’), KAT2A siRNA-3 (Sangon Biotech, 5’-CGAUGUUCGAGCUCUCAAA-3’), negative control siRNA (Sangon Biotech, 5’-UUCUCCGAACGUGUCACGUTT-3’). For KAT2A overexpression, cells were transiently transfected with pLV3‑CMV‑ 3 × FLAG‑Puro and pLV3‑CMV‑KAT2A(human)‑3 × FLAG‑Puro (Shanghai Qiming Bio‑tech Co., LTD.) using Lipofectamine 3000 (Thermo Fisher Scientific, USA) according to the manufacturer's instructions. After transfection, cells were infected with SC19 as described above. After 4 h infection, cells were collected for western blotting analysis and flow cytometry. The knockdown and overexpression efficiency of KAT2A were assessed by western blotting analysis.

### Cell counting kit‐8 (CCK-8) assay

CCK-8 assay was performed with a CCK-8 kit (Beyotime, C0042) according to the manufacturer’s protocol. Briefly, hCMEC/D3 cells were seeded in 96-well plates at 2 × 10^4^ cells/well and then were transfected with either si-NC or si-KAT2A. After 48 h of incubation, the corresponding cells were pretreated with Nec-1, followed by SC19 infection for 4 h. Subsequently, 10 μL of the CCK-8 solution was added to each well. After 1 h of incubation, the absorbance at 450 nm was measured using a microplate reader (Bio-Rad, Japan). The cell proliferation rate was calculated as the absorbance ratio of treated cells to non-treated and non-infected cells.

### Animal experiments

WT C57BL/6 mice (total *N* = 153, each group *n* = 5 or 12) were intraperitoneally infected with 100 μL SC19 (2 × 10^8^ CFU) and PBS as blank control. To inhibit KAT2A activity in vivo, mice were intraperitoneally administered with MB-3 (10 mg/kg) once every 24 h for three consecutive times, and infection was performed at 24 h after the final injection. After 24 h infection with SC19, mice (total *N* = 45, each group *n* = 5) were sacrificed by cervical vertebrae dislocation and brain tissues were collected to determine the tight junction protein expression. Additionally, the survival curves of mice (total *N* = 108, each group *n* = 12) were recorded. Three independent experiments were performed.

### Liquid chromatography tandem mass spectrometry (LC–MS/MS)

hCMEC/D3 cells including si-KAT2A cells and si-NC cells were infected with SC19 for 4 h as described above. After infection, cells were harvested, pelleted and snap-frozen in liquid nitrogen. Samples were shipped on dry ice to APExBIO Technology LLC (Shanghai, China) for proteomic analysis. Protein extraction, digestion, and LC–MS/MS analysis were performed by APExBIO following their standard label-free 4D-DIA (data-independent acquisition with ion-mobility) workflow. Briefly, proteins were reduced, alkylated, and digested with trypsin, and the resulting peptides were desalted and analyzed by nano-LC coupled to high-resolution MS. Peptides were separated on a C18 reversed-phase column using a gradient of acetonitrile with 0.1% formic acid. DIA-MS data were acquired in positive ion mode using higher-energy collisional dissociation (HCD), enabling reproducible peptide identification and quantitative analysis.

### Statistical analysis

The experimental data were obtained from three independent experiments with three samples each group in vitro and five or twelve mice in vivo. Data were tested for normality and presented as the mean ± standard deviation (SD). For comparisons between two groups, an unpaired two-tailed Student’s *t* test was used. Comparisons among multiple groups were conducted using one-way ANOVA, followed by Tukey’s post-hoc test for multiple comparisons. For experiments involving two independent variables, two-way ANOVA was performed with Sidak’s post-hoc test. Kaplan–Meier survival curves were generated, and survival differences between groups were evaluated using the log-rank (Mantel-Cox) test. All statistical analyses were performed using GraphPad Prism 9 software. The *P*-values of less than 0.05 was defined as statistical significance.

## Results

### SC19 induces cell death in hCMEC/D3 cells

**Figure 1:**
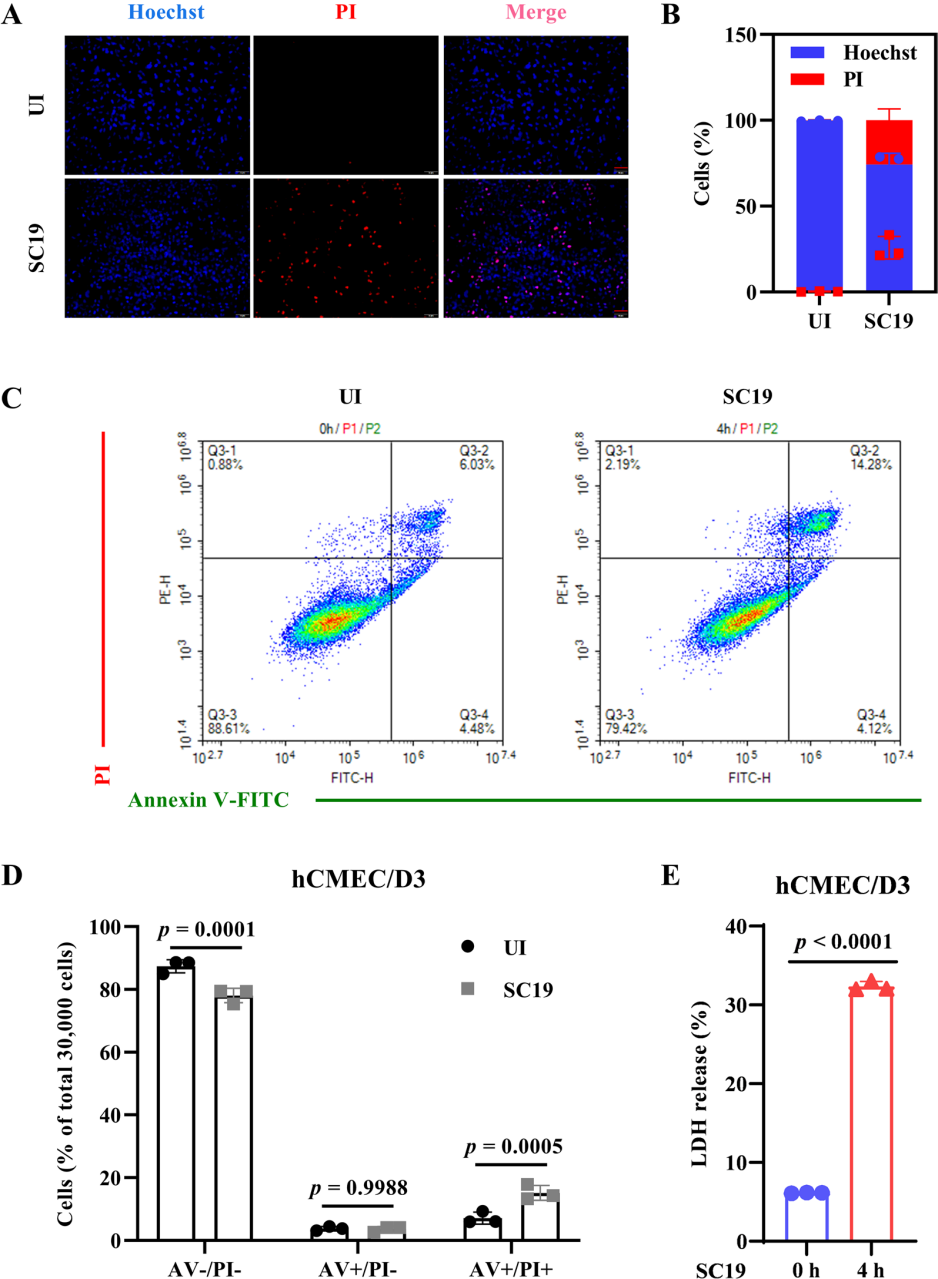
**SC19 induces cell death in hCMEC/D3 cells.** Cells were infected with SC19 at an MOI of 10 for 4 h, and cell death was subsequently determined. **A** Hoechst and Propidium iodide (PI) staining in SC19-infected cells. **B** Quantitative analysis of cell death. **C** Annexin V-FITC/PI staining detection using flow cytometry. **D** Quantitative flow cytometry analysis of Annexin V/PI staining. **E** Lactate dehydrogenase (LDH) release. All data were shown as mean ± SD and representative of three independent experiments, and analyzed using two-way ANOVA **(D)** or an unpaired two-tailed Student’s *t* test **(E)**. *P* ≤ 0.05 was considered statistically significant.

Brain microvascular endothelial cells are the first defense line of BBB and its disruption leads to the invasion of bacteria into CNS, thereby inducing meningitis. It has been reported that *S. suis* induces various programmed cell death including apoptosis [8, 10, 13, 28], pyroptosis [29], and necroptosis [12]. Therefore, we investigated whether *S. suis* strain SC19 induced cell death in hCMEC/D3 cells via PI staining, flow cytometry and LDH release. The results showed that SC19 significantly triggered PI influx in hCMEC/D3 cells with increased red fluorescent signal (Figures 1A and B), indicating that SC19 disrupted cell membrane and induced cell death. Additionally, SC19 significantly increased Annexin V +/PI + (late apoptotic/necrotic) populations (Figures 1C and D). Similarly, SC19 significantly induced LDH release in hCMEC/D3 cells (Figure 1E).

### SC19 induces the ubiquitin–proteasome-mediated degradation of KAT2A in hCMEC/D3 cells

**Figure 2:**
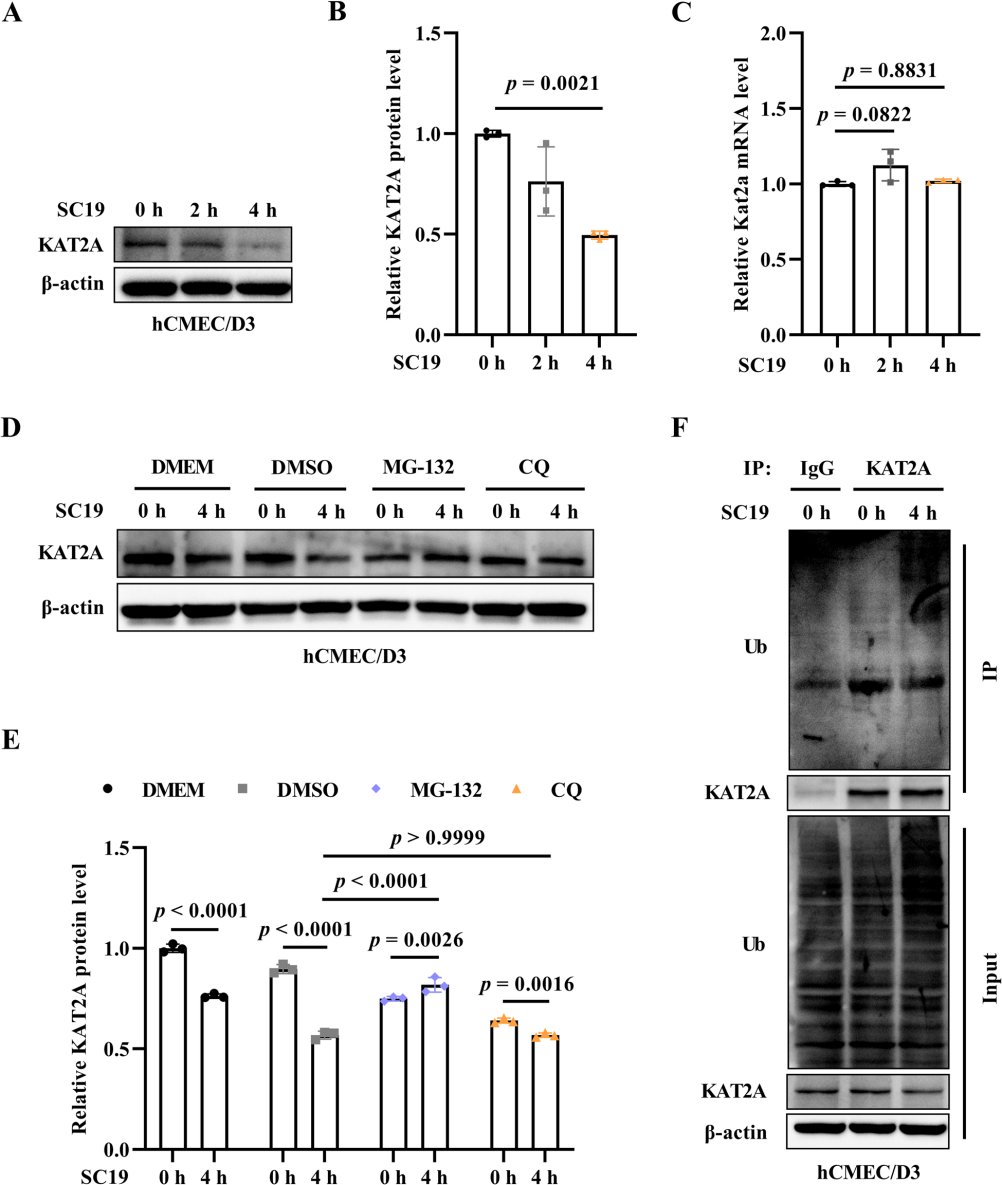
**SC19 induces the ubiquitin–proteasome-mediated degradation of KAT2A in hCMEC/D3 cells. A** Immunoblot analysis of KAT2A expression in hCMEC/D3 cells infected with SC19 for 0, 2, and 4 h. **B** Quantitative analysis of KAT2A protein expression level from (A). **C** mRNA expression of KAT2A in SC19-infected hCMEC/D3 cells for 2 and 4 h infection. GAPDH served as an internal control. **D** Immunoblot analysis of KAT2A in SC19-infected hCMEC/D3 cells under the treatment with either the proteasome inhibitor MG-132 or the lysosome inhibitor chloroquine (CQ). **E** Quantitative analysis of KAT2A protein expression level from (D). **F** Immunoblot analysis of ubiquitination of KAT2A in SC19-infected hCMEC/D3 cells using co-immunoprecipitation (Co-IP). All data were shown as mean ± SD and representative of three independent experiments, and analyzed using one-way ANOVA **(B, C)** or two-way ANOVA **(E)**. *P* ≤ 0.05 was considered statistically significant.

Next, upon SC19 infection, we observed that SC19 downregulated KAT2A protein expression in hCMEC/D3 cells in a time-dependent manner (Figures 2A and B) but did not affect mRNA expression (Figure 2C). To further investigate the mechanism by which SC19 induces degradation of KAT2A, MG-132 and CQ were used to inhibit ubiquitin–proteasome and lysosome pathway, respectively. As shown in Figures 2D and E, MG-132 reversed SC19-induced downregulation of KAT2A but CQ did not affect such downregulation. Furthermore, SC19 enhanced KAT2A ubiquitination at 4 h post infection (Figure 2F), indicating that SC19 targets degradation of KAT2A via ubiquitin–proteasome pathway.

### KAT2A exerts a protective role in maintaining blood–brain barrier integrity during SC19 infection in vitro and in vivo

**Figure 3:**
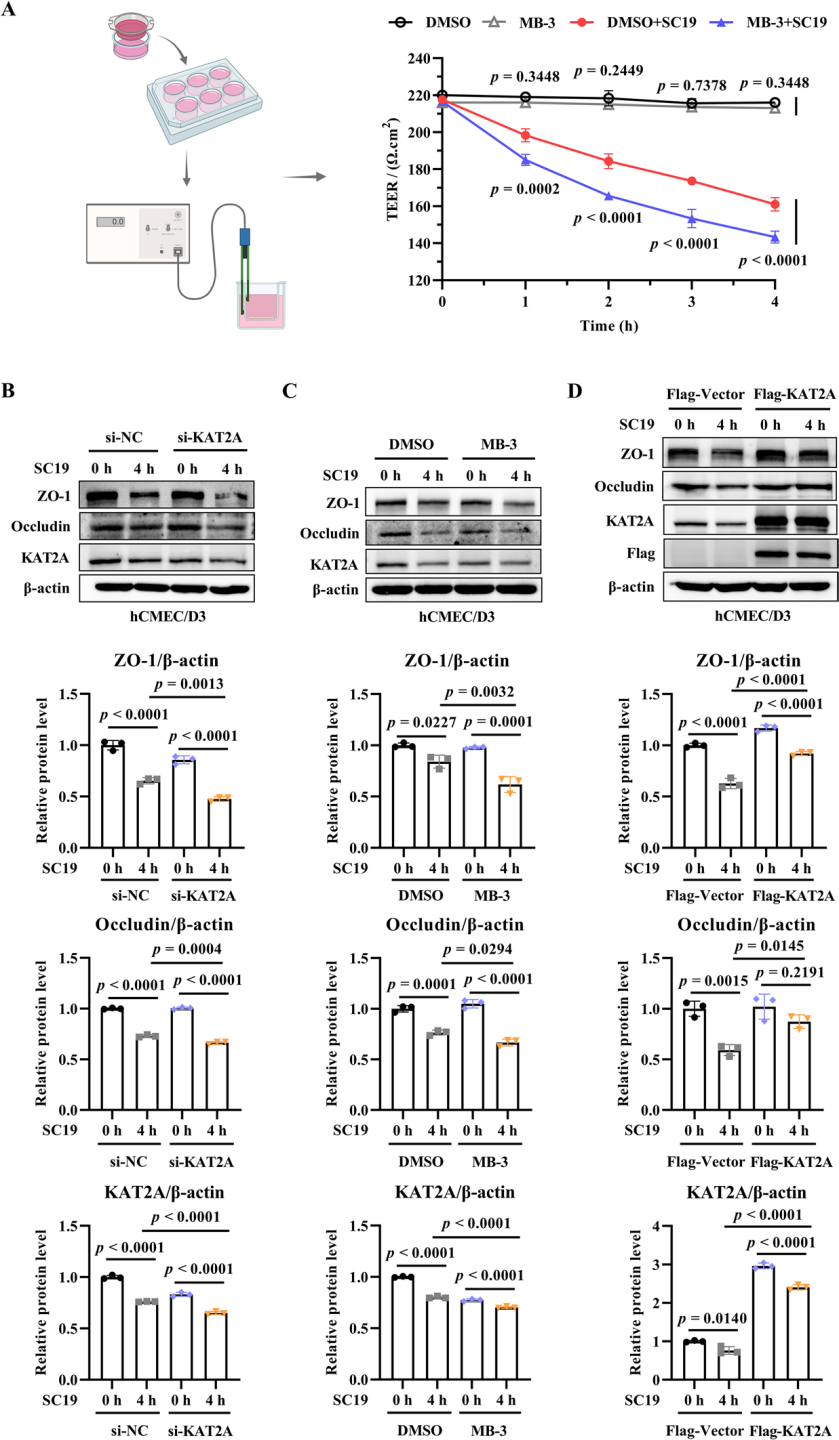
**KAT2A plays a protective role in maintaining BBB integrity during SC19 infection in vitro. A** The schematic diagram of SC19 infection transwell model was generated by BioRender (left panel). Then, TEER values were measured in SC19-infected hCMEC/D3 cells for indicated times under the treatment of MB-3 or DMSO (right panel). **B** Immunoblot analysis of ZO-1 and occludin expression in SC19-infected hCMEC/D3 cells transfected with si-NC or si-KAT2A. **C** Immunoblot analysis of ZO-1 and occludin expression in SC19-infected hCMEC/D3 cells treated with DMSO or MB-3. **D** Immunoblot analysis of ZO-1 and occludin expression in SC19-infected hCMEC/D3 cells transfected with pLV3-CMV-3 × FLAG-Puro or pLV3-CMV-KAT2A(human)−3 × FLAG-Puro. All data were shown as mean ± SD and representative of three independent experiments, and analyzed using two-way ANOVA **(A)** or one-way ANOVA **(B, C, D)**. *P* ≤ 0.05 was considered statistically significant.

To investigate the potential role of KAT2A in *S. suis*-induced BBB disruption, KAT2A inhibitor MB-3 was used in a BBB transwell model employing hCMEC/D3 cells. We found that MB-3 resulted in significantly lower TEER values compared to non-MB-3 treated cells during SC19 infection (Figure 3A), indicating that KAT2A protects endothelial barrier integrity. To genetically validate this finding, small interfering RNAs (siRNAs) were used to knockdown the expression of KAT2A (Additional file 1A), and a KAT2A expression construct (pLV3-CMV-KAT2A(human)−3 × FLAG-Puro) was used to overexpress KAT2A (Additional file 1B). The knockdown efficiency was verified by western blotting. The third siRNA (si-KAT2A-3) showed the highest knockdown efficacy and was selected for subsequent experiments (Additional file 1A). We further investigated the effect of KAT2A knockdown on the expression of tight junction proteins. The results showed that SC19 downregulated the expression of ZO‑1 and occludin, this reduction was further exacerbated by KAT2A knockdown (Figure 3B) and by pharmacological inhibition with MB‑3 (Figure 3C), whereas overexpression of KAT2A partially restored ZO‑1 and occludin levels in SC19-infected cells (Figure 3D).

**Figure 4:**
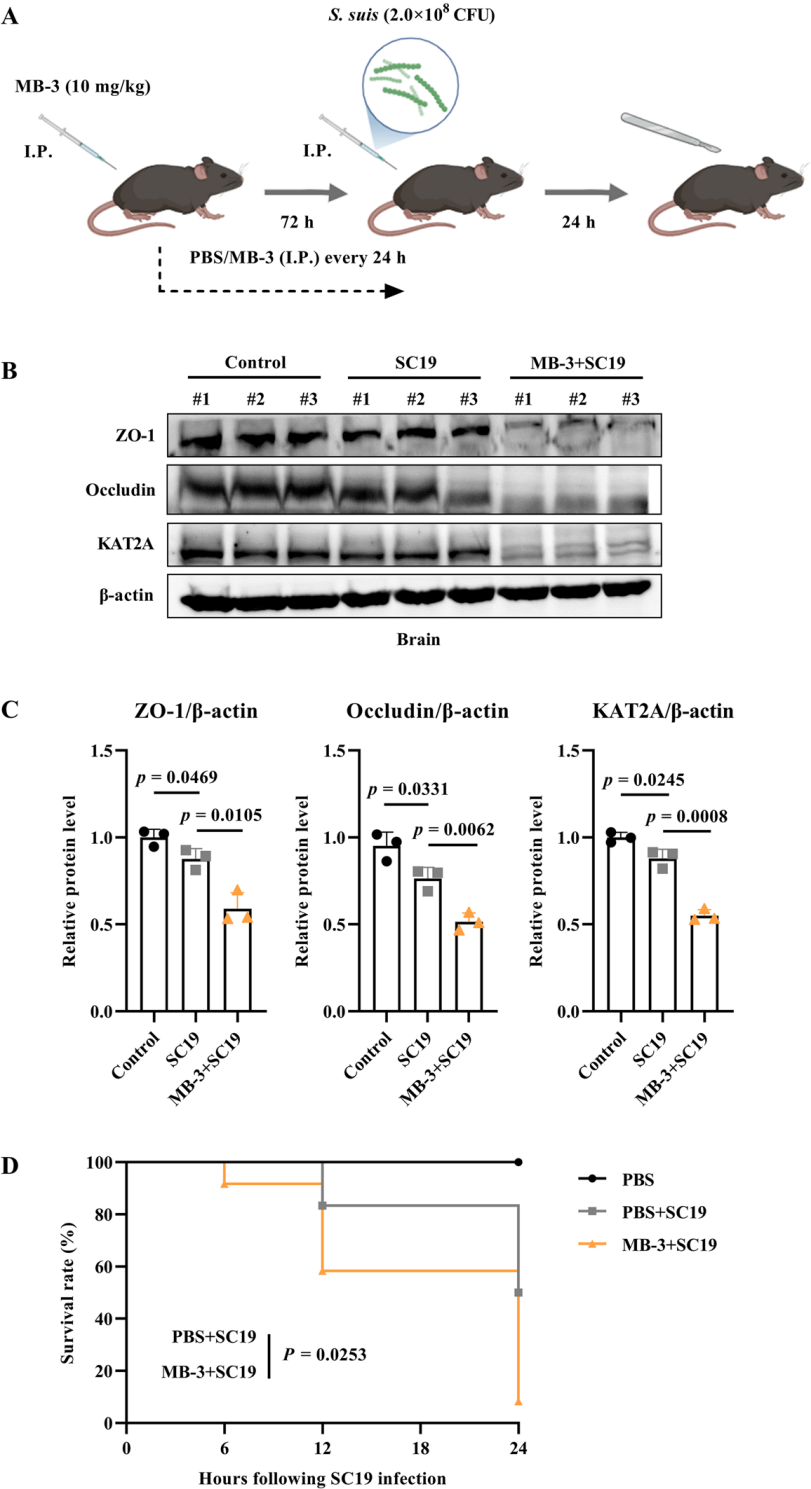
**Pharmacological inhibition of KAT2A reduces expression of tight junction proteins and increases mortality in SC19-infected mice. A** Schematic diagram of the experimental design for the in vivo mouse infection model. Mice were pretreated with PBS or the KAT2A inhibitor MB-3, followed by SC19 (2 × 10^8^ CFU) infection for 24 h. **B** Immunoblot analysis of ZO-1, occludin and KAT2A protein expression in brain tissues. **C** Quantitative analysis of ZO-1, occludin and KAT2A protein expression levels in brain tissues. **D** The survival rate of mice infected with SC19 in the presence and absence of MB-3 pretreatment. All data were presented as mean ± SD and representative of three independent experiments, and analyzed using an unpaired two-tailed Student’s *t* test **(C)** or the log-rank (Mantel-Cox) test **(D)**. *P* ≤ 0.05 was considered statistically significant.

Next, we further explored the role of KAT2A in SC19-induced BBB disruption in vivo using a mouse model. Figure 4A illustrates a schematic diagram that mice were pretreated with MB-3 followed by SC19 infection. The results showed that SC19 downregulated expression of KAT2A, occludin and ZO-1 and KAT2A inhibition using MB-3 enhanced such reduction in brain (Figures 4B and C). Furthermore, MB-3 treatment induced higher mortality. Notably, the survival rate of mice in the MB-3 + SC19 group dropped to 8.3% at 24 h post-infection, whereas the survival rate of mice in PBS + SC19 group was maintained at 50.0% (Figure 4D). These results demonstrate that KAT2A plays a protective role in maintaining BBB integrity during *S. suis* infection.

### KAT2A regulates SC19-induced necroptosis in hCMEC/D3 cells in a RIPK1-dependent manner

**Figure 5:**
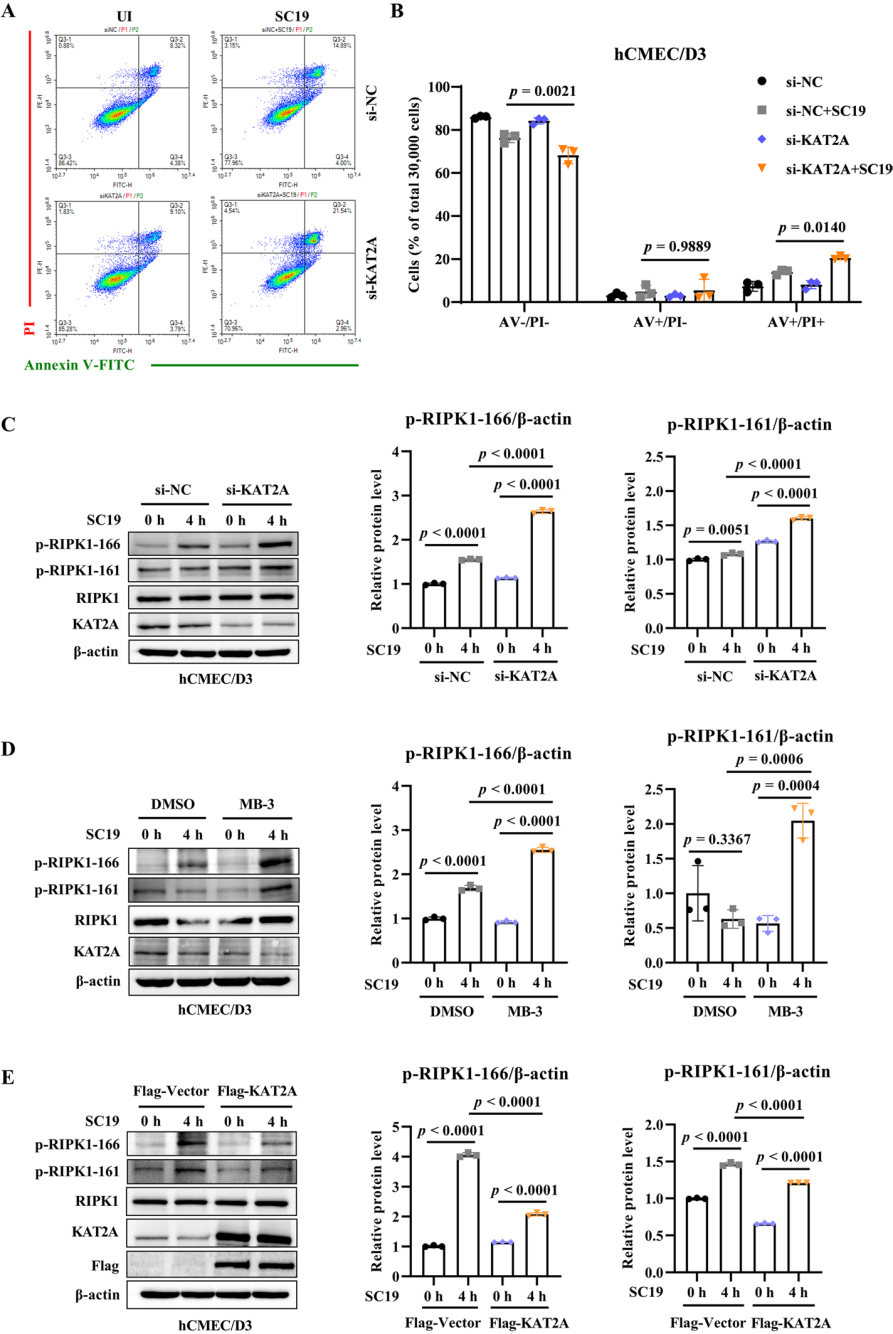
**KAT2A suppresses SC19-induced necroptosis and RIPK1 activation in hCMEC/D3 cells. A** Flow cytometry analysis of cell death using Annexin V-FITC/PI staining in SC19-infected hCMEC/D3 cells transfected with si-NC or si-KAT2A. **B** Quantitative analysis of the percentage of Annexin V-negative/PI-negative (AV-/PI-), Annexin V-positive/PI-negative (AV +/PI-) and Annexin V-positive/PI-positive (AV +/PI +) cells from three independent experiments. **C** Immunoblot analysis of phosphorylated RIPK1 (p-RIPK1 Ser166/Ser161) and RIPK1 in SC19-infected hCMEC/D3 cells transfected with si-NC or si-KAT2A. **D** Immunoblot analysis of phosphorylated RIPK1 (p-RIPK1 Ser166/Ser161) and RIPK1 in SC19-infected hCMEC/D3 cells treated with DMSO or MB-3.** E** Immunoblot analysis of phosphorylated RIPK1 (p-RIPK1 Ser166/Ser161) and RIPK1 in SC19-infected hCMEC/D3 cells transfected with pLV3-CMV-3 × FLAG-Puro or pLV3-CMV-KAT2A(human)−3 × FLAG-Puro. All data were shown as mean ± SD and representative of three independent experiments, and analyzed using two-way ANOVA **(B)** or one-way ANOVA **(C, D, E)**. *P* ≤ 0.05 was considered statistically significant.

**Figure 6:**
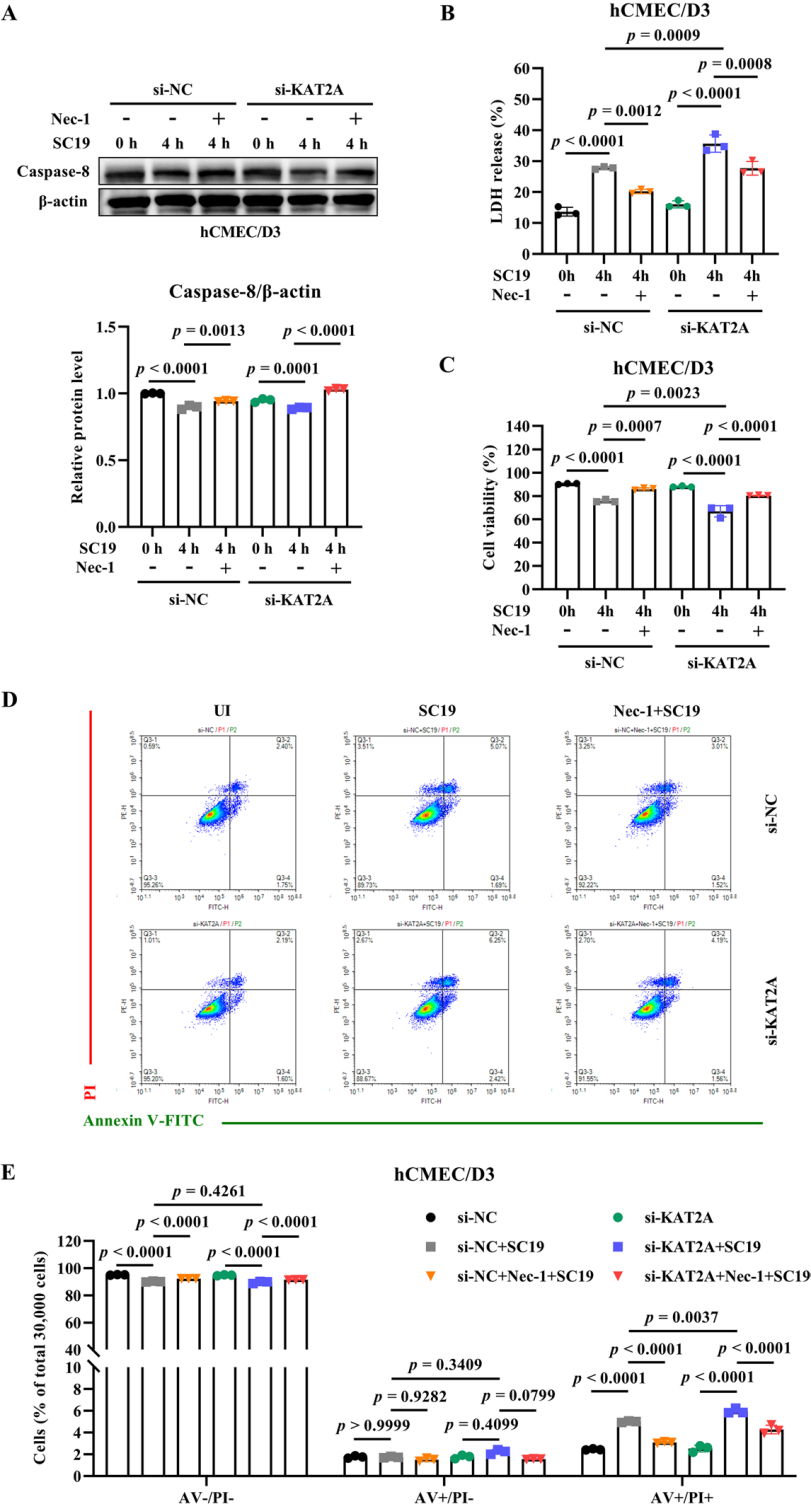
**KAT2A deficiency exacerbates SC19-induced necroptosis in hCMEC/D3 cells, which is reversed by Nec-1 treatment. A** Immunoblot analysis of caspase-8 expression in SC19-infected hCMEC/D3 cells transfected with si-NC or si-KAT2A in the presence and absence of Nec-1 treatment. **B** LDH release level in SC19-infected hCMEC/D3 cells transfected with si-NC or si-KAT2A in the presence and absence of Nec-1 treatment. **C** Cell viability using CCK-8 assay in SC19-infected hCMEC/D3 cells transfected with si-NC or si-KAT2A in the presence and absence of Nec-1 treatment. **D** Flow cytometry analysis of cell death using Annexin V-FITC/PI staining in SC19-infected hCMEC/D3 cells transfected with si-NC or si-KAT2A in the presence and absence of Nec-1 treatment. **E** Quantitative analysis of the percentage of Annexin V-/PI- (viable), Annexin V +/PI- (early apoptotic), and Annexin V +/PI + (late apoptotic/necroptotic) cells. All data were shown as mean ± SD and representative of three independent experiments, and analyzed using one-way ANOVA **(A, B, C)** or two-way ANOVA **(E)**. *P* ≤ 0.05 was considered statistically significant.

To further investigate the mechanism by which KAT2A mediates BBB integrity, we assessed programmed cell death in hCMEC/D3 cells. The results of Annexin V-FITC/PI staining showed that KAT2A knockdown significantly increased the percentage of Annexin V/PI-positive cells during SC19 infection, whereas the proportion of Annexin V-positive/PI-negative (AV +/PI-) (early apoptotic) cells remained unchanged, indicating that KAT2A knockdown exacerbates SC19-induced necroptosis in hCMEC/D3 cells (Figures 5A and B). Consistently, pharmacological inhibition of KAT2A with MB-3 significantly increased LDH release in SC19-infected cells (Additional file 1C), whereas KAT2A overexpression effectively attenuated SC19-induced LDH release (Additional file 1D). Furthermore, we found that SC19 induced the phosphorylation of RIPK1 at Ser166 and Ser161 (p-RIPK1) (Figures 5C-E), indicating the activation of necroptosis. KAT2A knockdown and pharmacological inhibition with MB-3 significantly upregulated SC19-induced RIPK1 phosphorylation (Figures 5C and D). Conversely, KAT2A overexpression effectively attenuated the phosphorylation of RIPK1 during SC19 infection (Figure 5E). In addition, SC19 downregulated the expression of caspase-8, which is a key mediator of apoptosis and also exerts as a negative regulator of necroptosis since its inhibition relieves the suppression of RIPK1 signaling to promote necroptosis activation. KAT2A knockdown downregulated caspase-8 expression but RIPK1 inhibitor Nec-1 reversed its downregulation in KAT2A-knockdown cells (Figure 6A). Similarly, KAT2A knockdown significantly increased LDH release in SC19-infected cells while Nec-1 pretreatment rescue this increase in LDH release (Figure 6B). In addition, KAT2A knockdown remarkably reduced cell viability in SC19-infected cells and Nec-1 pretreatment reversed this reduction (Figure 6C). Consistently, Nec-1 treatment significantly reduced the proportion of AV +/PI + cells in both SC19-infected cells and KAT2A-knockdown cells (Figures 6D and E). Together, these findings suggests that SC19 infection triggers necroptosis in hCMEC/D3 cells and KAT2A regulates necroptosis through modulating RIPK1 activity.

### The proteomic landscape in KAT2A knockdown hCMEC/D3 cells

**Figure 7:**
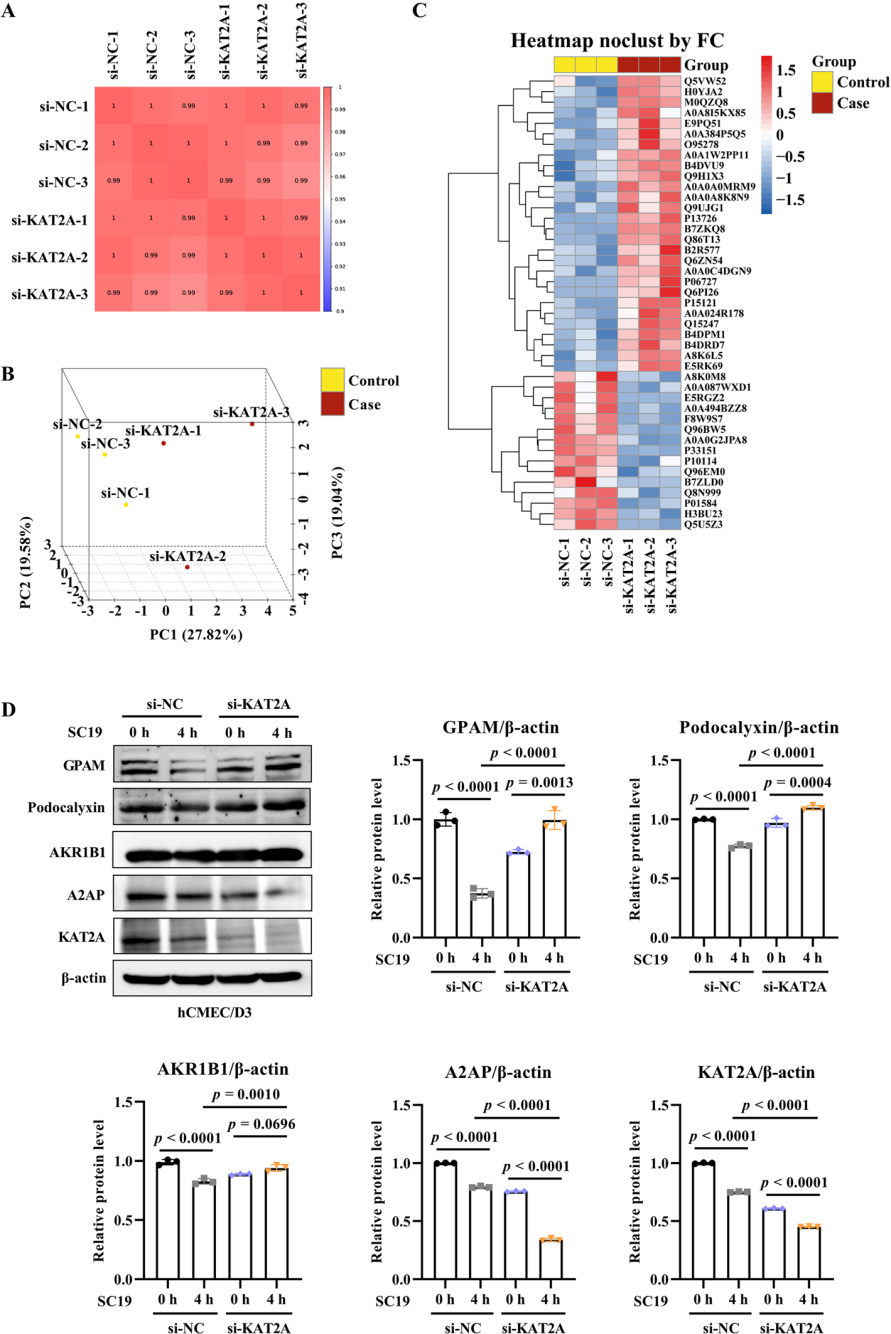
**Quantitative proteomic characterization in SC19-infected hCMEC/D3 cells in response to KAT2A knockdown. A** Pearson's correlation coefficient (PCC) heatmap of all samples from si-NC or si-KAT2A cells infected SC19. **B** Principal component analysis (PCA) plot showing clear separation between the si-NC and si-KAT2A groups. **C** Heatmap of top differentially expressed proteins (DEPs). **D** Immunoblot validation of representative DEPs including GPAM, Podocalyxin, AKR1B1 and A2AP. All data were shown as mean ± SD and representative of three independent experiments, and analyzed using one-way ANOVA **(D)**. *P* ≤ 0.05 was considered statistically significant.

To further investigate the mechanism of KAT2A-mediated BBB integrity during SC19 infection, we performed quantitative proteomics (4D-DIA LC–MS/MS) in KAT2A knockdown hCMEC/D3 infected with SC19. High data quality was evident by strong intra-group reproducibility in pearson correlation analysis (PCC) (Figure 7A) and principal component analysis (PCA) revealed that si-KAT2A and si-NC cell groups were clearly separated by the proteome level (Figure 7B). We identified 43 differentially expressed proteins (DEPs) between si-KAT2A and si-NC cells infected with SC19 based on a two-tailed Student’s *t*-test (*P* < 0.05) and the fold change (FC > 1.2). Among these DEPs, si-KAT2A cells exhibited 28 upregulated DEPs and 15 downregulated DEPs (Figure 7C). Gene Ontology (GO) and Kyoto Encyclopedia of Genes and Genomes (KEGG) analysis revealed that DPEs were significantly enriched in the biological processes and pathways related to cell–cell junction organization, regulation of apoptosis, inflammatory response, complement and coagulation cascades, and neurodegeneration (Additional files 2A and B).

In addition, subcellular localization analysis revealed that DEPs were significantly enriched in the plasma membrane, indicating the potential effect of KAT2A on maintaining cell barrier. Next, we investigated the expression of DEPs enriched in the regulation of inflammatory responses, cell fate and cell integrity. The results showed that KAT2A knockdown significantly upregulated the expression of glycerol-3-phosphate acyltransferase (GPAM) and podocalyxin (Figure 7D), which have been reported to regulate membrane permeability in cancer cells [30] and endothelial cells [31]. Aldo–keto reductase family 1 member B1 (AKR1B1) has been reported to protect against neuronal damage by counteracting chemical-induced neuronal apoptosis [32]. AKR1B1 expression is upregulated when cells are exposes to the oxidative stress conditions which are a common pathological trigger of neuronal injury. Similarly, KAT2A knockdown slightly induced AKR1B1 upregulation (Figure 7D), indicating that KAT2A deficiency may aggravate cell damage. Alpha-2-antiplasmin (A2AP), a serine protease inhibitor, plays a vital role in regulating the over-cleavage and release of vascular endothelial growth factor (VEGF) associated with BBB dysfunction [33]. Conversely, KAT2A knockdown induced A2AP downregulation (Figure 7D), indicating that KAT2A deficiency augments BBB dysfunction.

**Figure 8:**
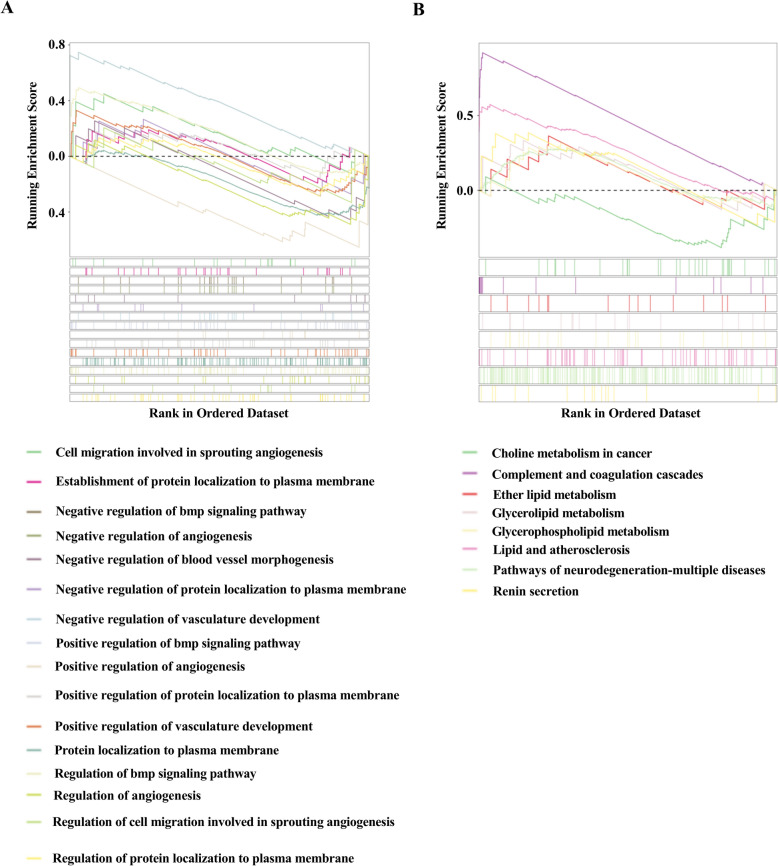
**Gene set enrichment analysis (GSEA) reveals pathway**-**level alterations induced by KAT2A knockdown. A** GSEA based on Gene Ontology (GO) terms. **B** GSEA based on Kyoto Encyclopedia of Genes and Genomes (KEGG) pathways.

Gene set enrichment analysis (GSEA) based on genome-wide gene expression profiles provided an unbiased at the pathway level, confirming that gene sets were significantly enriched in necrosis, apoptotic signaling pathway, and negative regulation of intracellular adhesion in the si-KAT2A cells infected with SC19 (Figure 8). Collectively, KAT2A deficiency reshapes the proteomic landscape of brain endothelial cells, especially toward enhanced cell death signaling and impaired barrier integrity during *S. suis* infection.

## Discussion

It is well known that BMECs are the important component of BBB and infection with *S. suis* disrupts the structural integrity of BBB. In this study, we reported a key mechanism by which *S. suis* disrupted BBB via inducing hCMEC/D3 cells necroptosis through downregulating KAT2A. The genetic knockdown and pharmacological inhibition of KAT2A aggravated the disruption of hCMEC/D3 cells integrity and necroptosis, whereas its overexpression effectively attenuated these effects, indicating its protective role in the BBB. Specifically, we observed that KAT2A inhibition or knockdown significantly reduced the expression of tight junction proteins and promoted RIPK1‑mediated necroptosis, both of which directly contributed to the increased permeability and decreased TEER of the blood–brain barrier. Our study reveals that KAT2A plays an essential role in the maintenance of the BBB integrity during *S. suis* infection.

KAT2A, an acetyltransferase that couples with α-ketoglutarate dehydrogenase (α-KGDH) and acetyl-CoA synthetase 2 (ACSS2), induces histone succinylation and lactylation to regulate gene expression, tumor cell proliferation and tumor development [14, 19]. The ability to induce specific protein posttranslational modifications plays a critical role in regulating the development of neuronal cells. For example, KAT2A induces tubulin acetylation to promote axon growth and neurogenesis [34]. KAT2A acetylates transcription factor PAX6 and promotes its ubiquitination, thereby modulating neural stem cell differentiation and proliferation [35]. KAT2A inhibition leads to apoptosis of neural stem cells. Similarly, our study demonstrated that *S. suis* infection induced KAT2A ubiquitination degradation. KAT2A knockdown promoted *S. suis*-induced BBB disruption, indicating the defensive function of KAT2A in the BBB against bacterial infection. Upon viral infection, KAT2A succinylates histone H3K79 in hepatitis B virus (HBV) cccDNA minichromosome and acetylates nucleoprotein (NP) of influenza A virus to promote viral replication [25, 36]. Apart from inducing pathogens’ protein modification, KAT2A also acetylates the host protein to regulate immune response against viral infection. It has been reported that KAT2A acetylates cGAS at lysine residues 421, 292, and 131 to trigger cGAS signaling activation and type I interferon production [37]. Furthermore, KAT2A regulates interferon signaling to maintain intestinal health [38]. Therefore, we speculate that KAT2A-mediated BBB integrity may be regulated by succinylation or acetylation of proteins of the host or bacteria induced by KAT2A, but the exact post-translational modification protein and its action of mechanism on the BBB need to be further studied.

In metabolic diseases, KAT2A expression is upregulated and its inhibition effectively ameliorates ferroptosis by mediating histone succinylation [21, 22]. However, our study showed that KAT2A expression was downregulated by *S. suis* and KAT2A knockout augmented RIPK1-mediated necroptosis in *S. suis*-infected hCMEC/D3, suggesting a regulatory role of KAT2A on cell death. It has been reported that *S. suis* virulence factors such as peptidyl-prolyl isomerase (PrsA), suilysin and enolase induce cell death including pyroptosis, necroptosis and apoptosis [8, 10, 12]. Therefore, whether KAT2A-mediated acetylation or succinylation of these virulence factors regulates cell death remains to be further investigated. Notably, during *S. suis* infection, NLRP3 inflammasome is activated in hBMECs [7] and involved in BBB disruption. In inflammatory diseases, KAT2A is increased and induces metabolic reprogramming in macrophage to regulate NLRP3 inflammasome activation and it is reported as a potential therapeutic target [15]. *S. suis*-induced KAT2A downregulation suggests that this reduction may disrupt its regulatory function over NLRP3 inflammasome activation, thereby exacerbating BBB disruption.

The 4D-DIA proteomics analysis identified 43 DEPs in KAT2A knockdown hCMEC/D3 during SC19 infection. We selected GPAM, podocalyxin, AKR1B1 and A2AP for orthogonal validation because they functionally regulate barrier integrity or stress responses [31, 39–42]. Although *S. suis* abrogated KAT2A function via downregulating its expression, KAT2A knockdown may trigger functional compensation of other upregulated or downregulated proteins, which could either exacerbate or alleviate *S. suis*-induced BBB disruption. Notably, KAT2A knockdown specifically upregulated the expression of GPAM and podocalyxin. GPAM is critical for phospholipid synthesis, thereby affecting membrane microdomain organization and junctional stability [40]. Meanwhile, podocalyxin is known to be essential for maintaining BBB integrity under inflammatory stress [31]. Such upregulation of GPAM and podocalyxin indicates a host compensatory defensive response to the loss of KAT2A-mediated protective functions. On the other side, A2AP is the primary inhibitor of plasmin and its reduction leads to severe sepsis during bacterial infection [42]. In line with this, our observation of A2AP reduction in the current study suggests that KAT2A deficiency leads to attenuation of A2AP’s protective effects, which may ultimately exacerbate *S. suis*-induced BBB disruption. However, the exact mechanism by which KAT2A mediates the expression of these proteins to regulate BBB integrity remains to be further elucidated.

In conclusion, our study demonstrates that the degradation of the epigenetic regulator KAT2A represents a pivotal mechanism by which *S. suis* SC19 impairs endothelial barrier defenses, promotes necroptosis, and consequently facilitates BBB breakdown. Future work should focus on identifying the E3 ubiquitin ligase(s) mediating KAT2A ubiquitination and the specific substrates of KAT2A-induced acetylation. Such investigations will further elucidate the mechanism by which KAT2A regulates *S. suis*-induced BBB disruption. These efforts will enhance the potential for translating these findings into pharmacological interventions to treat bacterial meningitis.

## Supplementary Information

**Additional file 1:** **A Assessment of KAT2A knockdown efficiency by siRNA in hCMEC/D3 cells.** **B** Validation of KAT2A overexpression efficiency in 293 T cells. Cells were transfected with the pLV3-CMV-KAT2A-3×FLAG-Puro plasmid or an empty vector. **C** LDH release level in SC19-infected hCMEC/D3 cells treated with DMSO or MB-3. **D** LDH release level in SC19-infected hCMEC/D3 cells transfected with the pLV3-CMV-KAT2A-3×FLAG-Puro plasmid or an empty vector.

**Additional file 2:** **Functional classification and subcellular localization of differentially expressed proteinsupon KAT2A knockdown. A **GO enrichment of DEPs in biological processes. **B** KEGG pathway enrichment of DEPs. **C** Subcellular localization classification enrichment of DEPs.

## Data Availability

All data supporting the findings of this study are available within the paper, including Supplementary Information. Supplementary Information contains Additional files 1, 2 and original full length western blots. Biological materials (strains, antibodies) are available from the corresponding author on reasonable request. Correspondence and requests could be addressed to rdfang@swu.edu.cn. The mass spectrometry proteomics data have been deposited to the ProteomeXchange Consortium [43] via the iProX partner repository with the dataset identifier PXD070383.
