# Supplementary figures and images for: Streptococcus suis disrupts the blood–brain barrier through inducing ubiquitin–proteasome-mediated degradation of KAT2A
Source: Vet Res. 2026 Mar 25;57:60. doi: 10.1186/s13567-026-01736-8 (PMC13141353; doi:10.1186/s13567-026-01736-8)

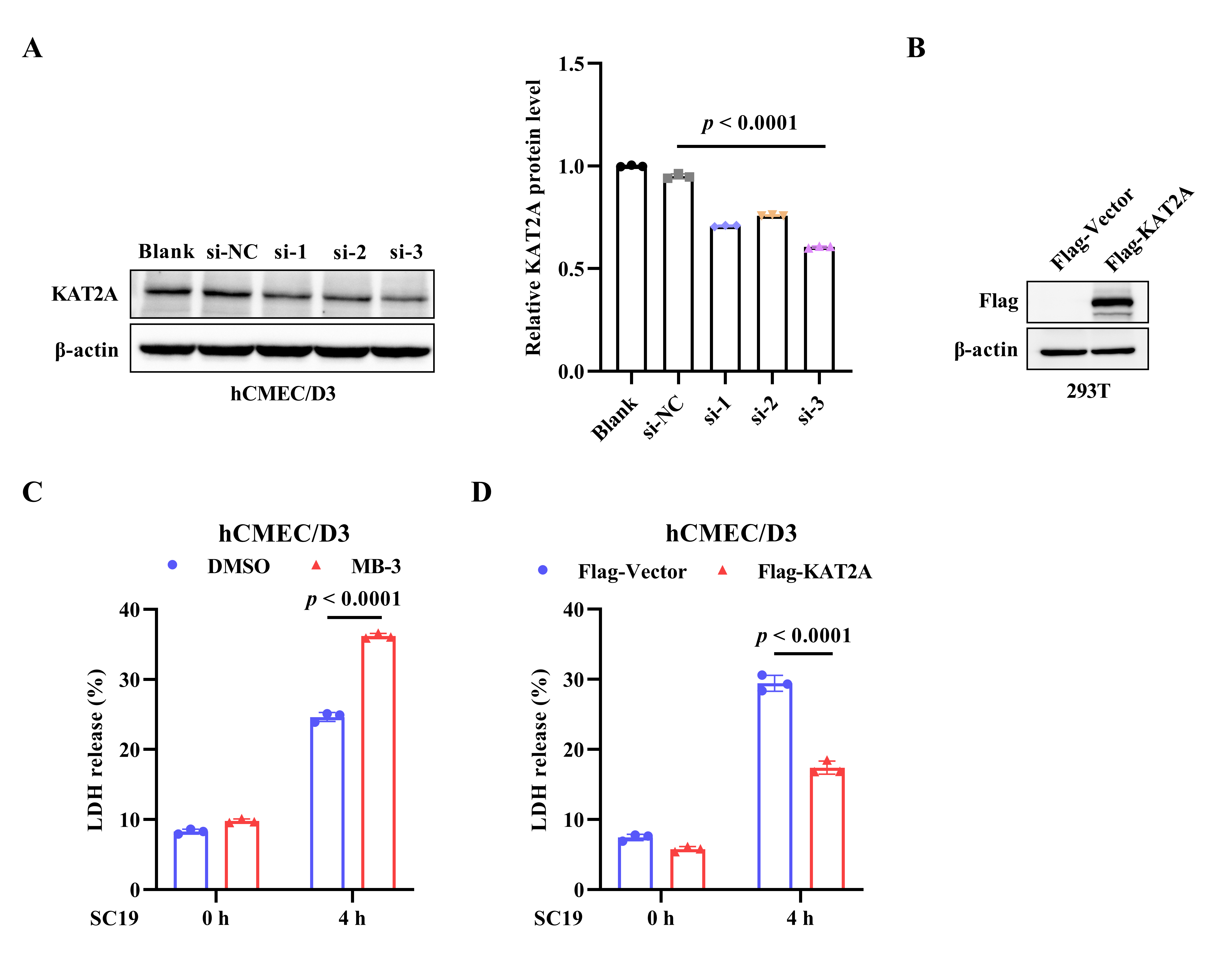

Supplement: Supplementary file 1 — Additional file 1: A Assessment of KAT2A knockdown efficiency by siRNA in hCMEC/D3 cells. B Validation of KAT2A overexpression efficiency in 293 T cells. Cells were transfected with the pLV3-CMV-KAT2A-3×FLAG-Puro plasmid or an empty vector. C LDH release level in SC19-infected hCMEC/D3 cells treated with DMSO or MB-3. D LDH release level in SC19-infected hCMEC/D3 cells transfected with the pLV3-CMV-KAT2A-3×FLAG-Puro plasmid or an empty vector. [file 13567_2026_1736_MOESM1_ESM.tif]

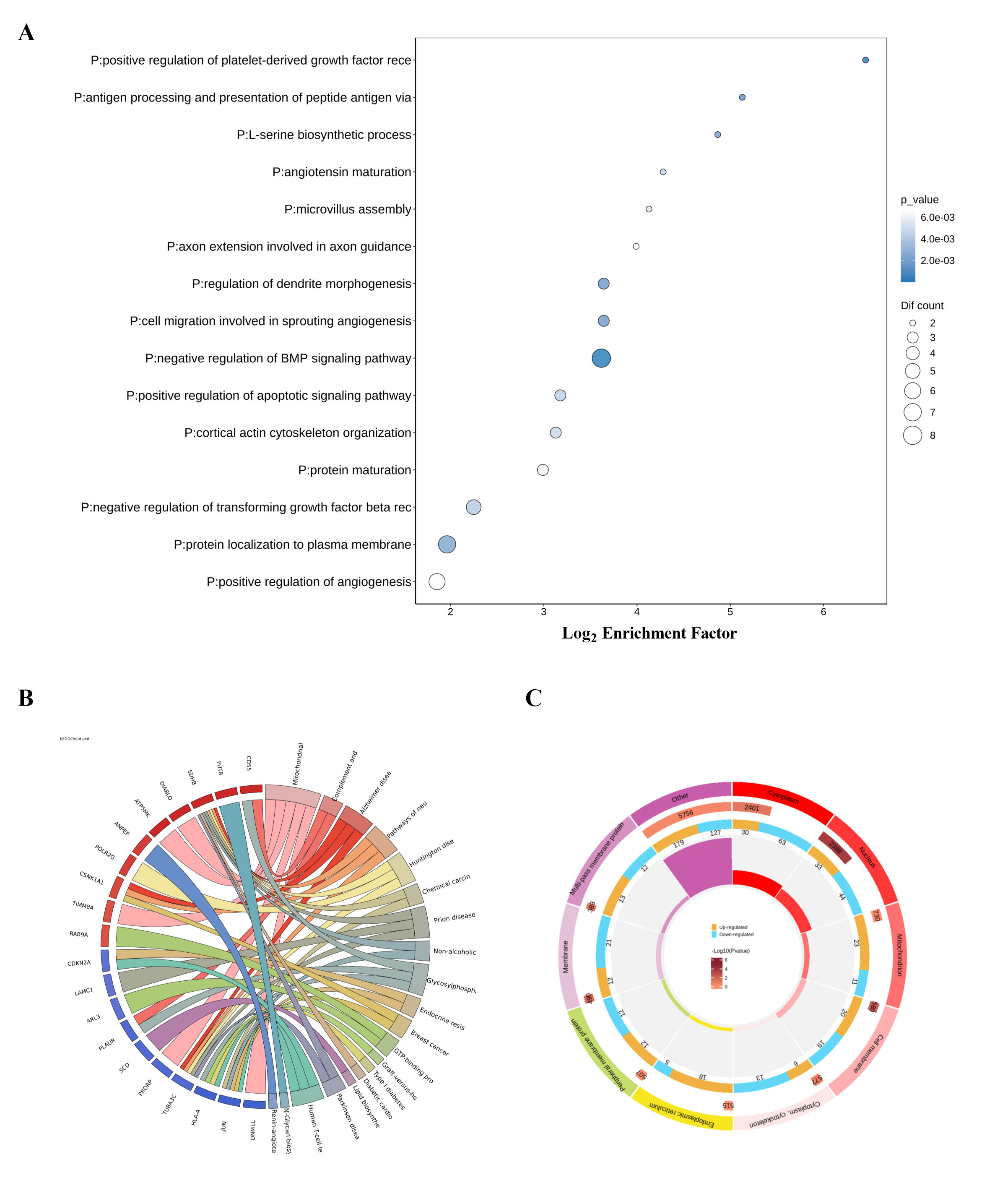

Supplement: Supplementary file 2 — Additional file 2: Functional classification and subcellular localization of differentially expressed proteinsupon KAT2A knockdown. A GO enrichment of DEPs in biological processes. B KEGG pathway enrichment of DEPs. C Subcellular localization classification enrichment of DEPs. [file 13567_2026_1736_MOESM2_ESM.tif]
